# Biological removing of Cadmium from contaminated media by fungal biomass of *Trichoderma* species

**DOI:** 10.1186/2052-336X-12-102

**Published:** 2014-07-04

**Authors:** Fariba Mohsenzadeh, Farzad Shahrokhi

**Affiliations:** 1 Department of Biology, Faculty of Science, Bu-Ali Sina University, Hamedan, Iran; 2 Department of Environment, Islamic Azad University, Hamedan Branch, Hamadan, Iran

**Keywords:** Biological removing, Cadmium, Heavy metals, *Trichoderma* fungi

## Abstract

**Background:**

Environment pollution by heavy metals is a global disaster and there are several cleaning methods including bioremediation. *Trichoderma* species inhibit the growth of pathogenic fungi and play a useful role in agriculture and ecosystem management.

**Methods:**

In this study, the removing of cadmium ions by three species of *Trichoderma* (*T. asperellum*, *T. harzianum* and *T. tomentosum*) were studied under different pH (5, 7, 9) and different concentrations of Cd (1, 100, 200 ppm) in liquid media containing potato extract and dextrose. Above mentioned fungal strains were cultured in the Cd-polluted media and the remaining amount of metal ions in the media was measured after two months growth, using atomic absorption.

**Results:**

Results showed that all three fungal species were able to reduce the amount of Cd in the all three pH of the medias; but their removal ability varies depending on the species and cultural conditions. *T. asperellum* was showed maximum removal efficiency of cadmium (76.17%), (10.75 mg/g, at fungal dry weight). Based on our results, the most removal efficiency of Cd ions for the fungal species was evaluated in the alkaline pH.

**Conclusions:**

*Trichoderma* species are important fungi in decreasing of Cadmium ions. They have bioremediation potency under various pH and concentration conditions.

## Background

Environmental pollution with metals, semi-metals and organic contaminants is a serious problem of worldwide and contamination with heavy metals is one of the most dangerous pollutants [1]. Existence of heavy metals showed adverse effects on flora, fauna and cause to groundwater contamination through leaching. It is also causes to reduce the performance and product quality in agriculture and is dangerous for public health and other living organisms [2]. Also existence of heavy metals in the soil caused the environmental stresses that can lead to reduction of plant growth [2,3]. Heavy metals are emitted into the environment by sewage and waste materials from various resources such as metal plating, paint industry, metallurgy, released oil ingredients in the soil, combustion of fossil fuels, mining, ores washing, pesticides, colored material, batteries, natural erosion of rocks and so on [4]. Due to the toxic effects of these compounds particularly intervention on cellular enzymatic systems, are able to impose their biological effects at the cellular level and causes to severe detrimental effects on gastrointestinal, respiratory and nervous organs and tend to cell necrosis in the exposed organs [5]. When the heavy metals accumulate in the soil, they are affected microbial activity and can also put human health at risk; because they are entered in the food chain. Toxic effects of metals appear in the various processes such as reduction of nitrogen fixation, irregularities in synthesis of enzymes and entering into food chain [6].

There are various processes to reduce concentrations of heavy metals in the environment. In this regard, it is notable that the microorganisms use these contaminants as a source of nutrients and energy and convert them into soluble substances. This process is known as bioremediation [7]. In this method, there is the possibility of removing of one or more pollutants from environment, with a low cost, and remaining products have not detrimental effects on the ecosystem of contaminated sites [3]. The using of microorganism such as algae, fungi, bacteria, and yeasts that can absorb the heavy metals, have been considered by some prior researchers for bioremediation of heavy metal polluted media [1,8,9]. In this way, microorganisms immobile metal ions by means of linking them with their cell walls [10,11].

Selection of a biomass for using in bioremediation is very important, it should be abundance in environment and adapted to environmental conditions [12-15]. Many contaminants can be reduced by means of biological methods and some of them can be remediated by fungi [15]. In this regard, *Aspergillus niger* was used for remediation of silver and *Talarmyces emersonii* and *Basidiomycetes* were used for accumulation and recycling of uranium [16,17].

Some researchers were able to remove Cd, Ni and Pb by biological removal using fungi with the efficiency of 94.47% for Cd, 79.81% for Ni and 99.73% for Pb [18]. In another research, chromium and nickel uptake by resistant bacteria and fungi was studied and results showed that removal efficiency of nickel was 90% with the amount of 0.1 milligrams of fungal biomass of *Aspergillus niger*[19]. Also in other study, 0.7 g/l of fungal biomass of *Aspergillus niger* showed 84% removal of cadmium ions [20,21].

The *Trichoderma* fungal species are found commonly in the all types of soils and some of them have the ability for cleaning of polluted environments and can be applied as effective microorganisms for bioremediation of pollutants [11].

Although there are several researches about bioremediation potency of fungi for Cd, but the most used fungi, *Aspergillus* species for example, are pathogens or produced toxins in the media. The aim of this research is to find a fungal species with high potential of bioremediation of Cd in aqueous media. We are evaluated *Trichoderma* species that are non-parasitic and non-pathogen fungi that are useful fungi for agriculture and recycling of biomass in nature. Based on our bibliographical studies there is not any prior research about Cd bioremediation using *Trichoderma* species.

## Methods

### Preparation of fungal species

Three different fungal species belonging to the genus *Trichoderma* was obtained from the Laboratory of Mycology, Faculty of Agriculture, Bu-Ali Sina University. The samples were transferred to PDA (Potato Dextrose Agar) sterile media for *in vitro* testing use. The samples were kept in the refrigerator at 4°C temperature, after the fungal biomass was reached to maximal growth. These colonies are suitable for transferring to new media and were used in our experiments as resource [22].

### Preparation of the liquid media

After fungal proper growth on the solid media, liquid media were prepared with the formula containing the amount of 250 g/l potato extract, 20 g/l of dextrose, 0.25 g/l of tetracycline antibiotic (to prevent bacteria growth) in three pH (5,7,9). Lactic acid and KOH (3%) were used for adjustment of the pH in the prepared media [23].

### Preparation of solutions, treatment and measurement

In this study, at the first, the stock solution of cadmium (1000 ppm) was prepared from cadmium nitrate (Cd (NO_3_)_2_ , 4 H_2_O) salts. Three pH including 5, 7, and 9, three concentrations including 1, 100, and 200 ppm, and three fungal species including *Trichoderma asperellum*, *T. harzianum* and *T. tomentosum* were selected for determination of optimal removing condition for cadmium. In each experiment, one the above-mentioned factors were chosen as variable and the others were kept as constant. Fungal biomass was removed from the media after two months. For this aim, the existing media were passed through the filter paper (Whatman No. 42) then they were centrifuged at 1500 rpm for 5 min. The debris was discarded and the supper homogeneous liquid, without fungal particles, was collected [24]. Then the remaining metal ions that exists in media, was determined using atomic absorption spectrophotometer and was compared with the metal concentration at the beginning of experiments using the statistical analysis. It is noteworthy that, all experiments were repeated in three replicates and the average was reported as the final result. After experiments, according to the metal uptake capacity, the optimal conditions were found for using in remediation of metal-contaminated environments.

### Determination of removal efficiency

The following equilibrium (1) was used for determination of metal removal efficiency by the studied fungi [25].

(1) $$R=\left(\frac{P_{0}-P_{e}}{P_{0}}\right)\times 100$$

In this equilibrium, R is the percentage of metal removal by the fungal biomass, P_0_ is the initial concentration of metal ions (ppm) and P_e_ is the final concentration of metal ions (ppm) in the experimental media.

### Determination of removal efficiency based on the dry weight of the fungi

To obtain the dry weight of the fungal biomass, were placed in a glass dish and was hold in the desiccator at 105°C for 48 h. The following equilibrium (2) was used for determination of the amount of metal adsorbed per dry weight of fungi [25].

(2) $$q=\frac{\left(C_{i}-C_{e}\right)\times V}{M}$$

In this equilibrium q is the metal absorbance based on dry weight of fungi (mg/g), C_i_ is the initial concentration of metal at the beginning of experiment (ppm), C_e_ is the metal concentration at the end of experiment (ppm), V is the volume of the solution (l) and M is fungal dry mass (g).

### Statistical analysis

In this study, independent variables are pH of the solution, different concentrations of cadmium and various species of the *Trichoderma*. The dependent variable is removal efficiency of Cadmium in each of the solution. The obtained data were analyzed using PASW (SPSS 18) software. For testing of normality and distribution of data, the Kolmogorov- Smirnov test with 95% confidence level was used. Two-way analysis of variance, ANOVA, was used for comparing of the means of obtained results [26].

## Results

### Effect of pH on Cd removing

**Figure 1:**
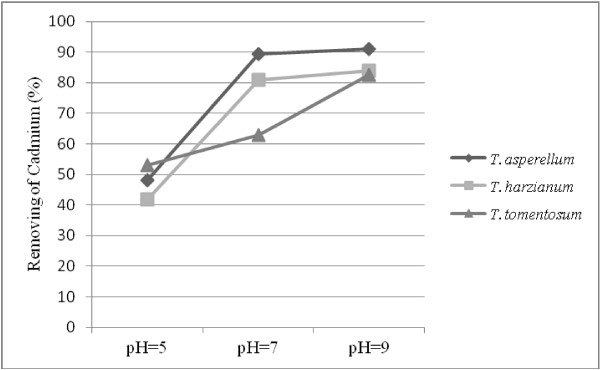
**Effect of pH on the removal ability of cadmium by *Trichoderma *species.** Maximum ability for cadmium removing was in pH = 9 for the all three *Trichoderma* species and the lowest removal ability was evaluated in pH = 5

The effect of pH on the Cd removing by the studied fungi in the comparison of blank samples (controls) was investigated in this study. According to Figure 1, maximum ability for Cadmium removing was in pH = 9 in the all three *Trichoderma* species. The lowest removal ability was evaluated in pH = 5. In pH = 7 removal capacity was considerably high, but it was lower than that of its amount at pH = 9. The optimal removing was obtained 91.06% (10.95 mg/g based on fungus dry weight) for *T. asperellum*, 83.92% (9.85 mg/g based on fungus dry weight) for *T. harzianum* and 82.63% (5.48 mg/g based on fungus dry weight) for *T. tomentosum* at the pH = 9. The all above-mentioned data represented decreasing of Cd in the experimental samples than control ones. After the reducing of pH to 5, removal ability of cadmium was decreased up to 48.14% (10.48 mg/g based on fungus dry weight) for *T. asperellum*, 41.84% (9.43 mg/g based on fungus dry weight) for *T. harzianum* and 53.04% (5.01 mg/g based on fungus dry weight) for *T. tomentosum*.

### Effect of concentration on Cd accumulation

**Figure 2:**
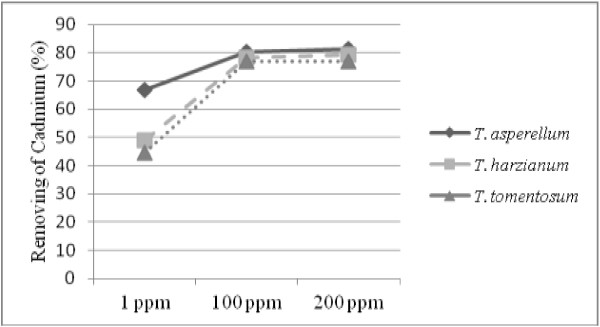
**Effects of concentration of Cd ions on the removal ability by *Trichoderma *species.** Results showed that with the increasing initial concentrations of Cd ions from 1 ppm to 100 ppm, adsorption capacity and removal ability of Cd was increased significantly (p ≤ 0.05).

Effect of initial concentration of Cd on the accumulation ability of the studied fungal species was studied. Results showed that Cd accumulation capacity in the fungal species was affected by its initial concentrations in the experimental media (Figure 2). According to the results, armed with the increasing initial concentrations of metal ions from 1 ppm to 100 ppm, adsorption capacity increased and removal ability of Cd was increased as following: For *T. asperellum* was increase from 66.88% to 80.37%, for *T. harzianum* increased from 40.0% to 78.35% and for *T. tomentosum* increased from 44.55% to 76.98%. In the initial concentration of more than 100 ppm, the significant change in the rate of metal removing was not observed and the difference between the absorption rate in the solutions with initial concentration of 100 ppm and 200 ppm was not significant (p ≤ 0.05).

### Comparing of the removal capacity of the *Trichoderma* species

**Figure 3:**
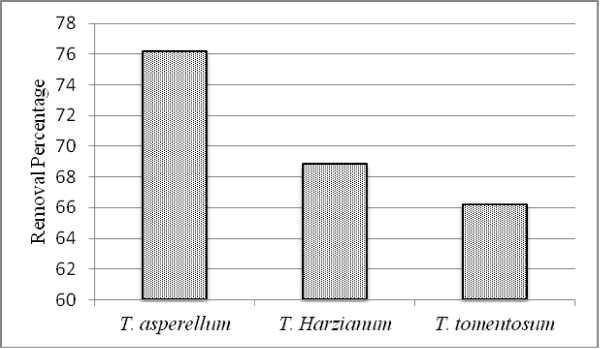
**Effect of *Trichoderma *species on the removal percentage of Cd ions.***T. asperellum* showed the highest ability and *T. tomentosum* showed the lowest ability in removal of Cd ions.

Comparing of metal removal capacity of the studied fungal species showed that there is a species-specific relationship between the fungi and Cd removing and then their removal ability (Figure 3 and Table 1). The results showed that *Trichoderma asperellum* has most ability in removal of cadmium ions (76.17%), (10.75 mg/g based on fungus dry weight) and *Trichoderma tomentosum* has the lowest ability in removal of Cadmium ions (66.19%), (5.23 mg/g based on fungus dry weight).

**Table 1:** **P-Value and F statistic values obtained for three different *Trichoderma *** **species in the 5% significance level**

**Species**	**Sources**	**Type III sum of squares**	**df**	**Mean square**	**F**	**Sig.**	**Partial eta squared**
***T. asperellum***	pH	680.10624	2	340.5312	879.1518	000.0	994.0
	Concentration	473.1169	2	737.584	185.167	000.0	949.0
	pH*Con.	583.23	4	896.5	686.1	197.0	273.0
	Error	956.62	18	498.3			
	Total	381.168578	27				
***T. harzianum***	pH	175.9919	2	588.4959	048.857	000.0	990.0
	Concentration	715.5348	2	358.2674	146.462	000.0	981.0
	pH*Con.	252.680	4	063.170	388.29	000.0	867.0
	Error	163.104	18	787.5			
	Total	573.144214	27				
***T. tomentosum***	pH	787.4085	2	894.2042	455.464	000.0	981.0
	Concentration	582.6322	2	291.3161	724.718	000.0	988.0
	pH*Con.	202.607	4	801.151	512.34	000.0	885.0
	Error	173.79	18	398.4			
	Total	709.129408	27				

^*^Interaction of pH and Concentration.

## Discussion

### Effect of pH on the Cd removing

pH is an important factor in the biological removing process and changes of pH, is very effective on chemical activity of metal ions that exists in solution and also absorption activity based on biomass and metal ions competition with each other [22].

In the of biological removing process, pH has impact two factors: first solubility of metal ions and the other total charge of the adsorbent, since the protons can be absorbed by the biomass or exert by it, in fungal case, this process depend on the functional groups on the surface of fungal cells and pH value of the media are effective on the balance of condition of the system [23]. The functional groups such as carboxyl, hydroxyl, amino and phosphate, on the fungal cell surfaces have major role in the uptake of Cd ions and they have different behavior at various pH conditions [24]. The effect of this factor will help us in determining the point at which biomass will maximum absorb. According to Figure 1, with increasing the amount of pH, adsorption capacity is also was increased. It is also indicated that in the low pH, with respect to cadmum nitrate that was used in this study, Cd ions are present in the form Cd^+2^, therefore this cations should be harder absorb to the biomass that absorbed proton and is acidified [17,27]. According to our results, the maximum absorption was evaluated at pH = 9. Similar result was reported by Das [28] about several strains of *Termitomyces clypeatus* in absorbing of Cd, Cu and Cr.

At the beginning of the experiment on the adsorption of heavy metals in aqueous solutions, increasing of pH is observed. This phenomenon could be justifiable according to the release of H^+^ from the some biomass components in solution that is a kind of ion exchange between H^+^ and metal ions. pH of a solution also affected by the chemical properties of the material and activity of functional groups (carboxylic, phosphate and amino) on the cell wall that they are in combination with metal ions in the desired position [29]. In general absorption behavior of various materials is similar, such as: (a) low absorption at the pH lower than 4 and (b) significant absorption when the pH of solution is in the range of 5 to 9 [9]. Of course the metal attachment to the cell wall is affected by pH, which is competing with other metal ions [6]. Lanouette [30] reported that in the initial concentration of 100 ppm and pH = 8, sedimentation of Nickel hydroxide ions is visible. This deposition reduced the amount of free nickel ions for the absorption and accumulation by organisms [30]. Akhtar et al. [10] was also demonstrated that carboxylic groups that are components of the cell wall of *Aspergillus niger*, are sites for metal ions absorption and in the very acidic pH values, the total surface charge of the cell is positive and thus metal cations and protons are competing for the sites in on the cell wall and tend to metal uptake reducing.

In the higher pH, carboxylic, phosphate and amino groups cause to increase the reaction of the metal ions, thus the effective adhesion is achieved quickly. In this pH, metal ions can move more quickly and more active sites are ready for the reaction with microorganisms. In pH lower than 3, functional groups in the cell wall occupy by H^+^ ions and limitation occurs for stand of metal ions [31]. Whereas with the increase of pH from 3 to 9, more functional groups, such as carboxylic and phosphate groups, become have negative charges and thus their ability is increasing or absorption of metal ions that have a positive charge [32]. Barros et al. [21] showed that removal percentage of Cd was about 37% in the solution with pH = 5 by the *Aspergillus niger* fungal biomass, that in compared with the amount in this study (48.01%), *Trichoderma* showed the better ability of metal removing in the favorable conditions regarding the initial concentration of metal ions and the suitable pH to absorption of heavy metals.

### Effect of initial concentration of cd ions on metal removal capacity

Absorption of metals by fungal biomass depends on their concentration. As is seen in the Figure 2, absorption of Cd ions increased by increasing of the initial concentrations and therefore the increasing the equilibrium concentration and it tend to increase the absorption force or concentration gradient [7]. After some increasing of uptake by increasing in initial concentration, absorption rate did not increased that could be due to the quickly occupation of adsorption sites (due to the high surface loading) caused by the high concentration which causes to reduce entrance of metal ions into the deep pores and thus the efficiency of absorption is reduced [4]. The similar result was reported Chergui [24] that used a kind of actinomycetes, *Streptomyces rimosus*, for removing of Cr, Cu and Z. The results showed by increasing the initial concentration more than 100 ppm, absorption rate does not increase and remained constant. This indicates that active sites become saturated on the surface of biomass. This issue can be justified that increasing the initial concentration increased collision between the absorber and metal ions partially and increased the absorption rate and it does not affected after saturation of absorption sites [22]. Leqba [27] and Sing [22] in their studies that have done on the absorption of Cu, Z and pb by *Phanerochaetechrys osporium*, showed that the increasing of initial concentration cause to increase the metals absorption that is due to increase of electrostatic interaction between metal ions and absorber sites.

Based on the some reports, the amount of adsorbed metal per unit mass of fungal biomass increased when the initial concentration of metal ions in solution was increased highly and it maybe a result of saturation of the active sites that leading to maximum adsorption capacity of metal ions by the biomass or due to the increase of the number of ions in competition for placement in the positions that existing in external wall of the biomass [28]. In the other words, adsorption capacity of the biomass increases with increasing of concentration of metal ions in solution [24]. In addition, increasing the concentration of metal ions causes to increase the number of collisions between metal ions and cell wall that tend to accelerate metal adsorption [7]. Results showed that, initial concentration of metal ions in 100 ppm of metal cause to absorb 82.24% of Cd. Some researchers [22,24] was studied the rate of Cd uptake by microorganism *Actinomycete* R 27 and results showed that the amount of adsorption was about 69%. Pradhan et al. [8] was studied the absorption rate of Chromium, Nickel and Iron and results showed that *Microcystis* was able to adsorb these metals in the range of 70% to 80%. The result of our study is accordance with the results of the above-mentioned reports. Jianlong et al. [23] was also observed 100% adsorption of Lead by the fungus *Aspergillus niger* at the concentration of 10 ppm. Their results indicated the high absorption of heavy metal ions by the organism, at low concentrations. Therefore, the limitation of metal ions uptake at low concentrations is less than at high concentrations of ions. With the considering the high pollution of sewage of the factories and high concentrations of water soluble metals, using the biomass of microorganisms especially the fungal biomass can be very useful for bioremediation of heavy metals [33-35].

Based on our bibliographical studies there is few report about Cd accumulation ability of *Trichoderma* species, just *T. harzianum* species [36]. Our finding data showed bioremediation ability of *Trichoderma* species and indicated that *T. asperellum* is the most effective species in removing of Cd*.* The most advantage of our finding is non-pathogenic properties of the studied fungi instead of prior studied fungi, *Aspergillus* species, for example.

## Conclusion

Biological absorption is an effective technology for the optimal removal of heavy metals [37,38]. In this process, adsorption and desorption rates are very high. Simplicity of the operation cause to introduce this technique as the best methods for removing of toxic materials from environment. *Trichoderma* species are the terricolous fungi that showed the absorption ability of heavy metals from aqueous solutions. At higher pH, the absorption is more and the highest absorption was in the pH = 9. At the lower pH competition occurs between metal cautions and H^+^ ions to connect to the fungal cell wall and the rate of absorption was reduced. One of the advantages of absorption by fungi is high-speed absorption. The pH and initial concentration was also effective on metal absorption rate. Based on our results the maximum Cd uptake is 76.17% that is evaluated for *T. asperellum* in the optimal conditions. This is the first report for the fungus.

## Competing interests

The authors declare that they have no competing interests.

## Authors’ contributions

FSH, is a MSc student and this manuscript was wrote based on her thesis results. He analyzed the samples and wrote initial manuscript. FM, is the supervisor of the thesis and supervised the methods and the project. She wrote the original research plan of the project and edited the manuscript. Both authors read and approved the final manuscript.
